# Steroid‐induced reconstitution of the biliary tree ravaged by IgG4‐related disease

**DOI:** 10.1002/ccr3.3109

**Published:** 2020-07-16

**Authors:** İlgin Özden, Arzu Poyanlı, Yasemin Sanlı, Sabahattin Kaymakoğlu

**Affiliations:** ^1^ General Surgery (Hepatopancreatobiliary Surgery Unit) Istanbul Faculty of Medicine Istanbul University Istanbul Turkey; ^2^ Radiology Istanbul Faculty of Medicine Istanbul University Istanbul Turkey; ^3^ Nuclear Medicine Istanbul Faculty of Medicine Istanbul University Istanbul Turkey; ^4^ Internal Medicine (Gastroenterology Unit) Istanbul Faculty of Medicine Istanbul University Istanbul Turkey

**Keywords:** autoimmune pancreatitis, biliary stricture, IgG4, perihilar cholangiocarcinoma, primary sclerosing cholangitis

## Abstract

The steroid‐induced, rapid healing of the biliary tree ravaged by IgG4‐related disease shows that the point of irreversibility remains to be defined.

## Graphical abstract

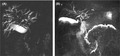

A 52‐year‐old man was referred for perihilar cholangiocarcinoma (Figure 1A). The available PET scan showed high activity at the perihilar mass and a discrete 1.7‐cm‐area in the right lobe (Figure 1B).

**Figure 1:**
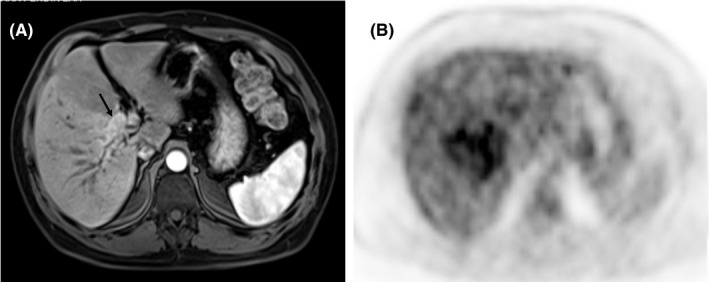
A, MRI showed a hypervascular perihilar mass obstructing the biliary tree (arrow), with more prominent dilation in the right lobe. B, High FDG uptake (SUVmax: 10.5) at the hilar mass and a discrete 1.7‐cm‐area in the right lobe (SUVmax: 8.6) (not visualized on the MRI)

The biochemistry results were as follows: AST:88 U/L, ALT:114U/L, ALP: 418 U/L (<105), GGT:383 U/L (5‐85); total bilirubin, CEA, and CA19‐9 were within normal limits. The MRCP taken at our institution showed a biliary tree ravaged by sclerosing cholangitis (Figure 2A). The IgG4 level was 1360 mg/dL (<201). The tests for ANA, ALKMA, AMA, ASMA, and p‐ANCA were negative. Oral prednisolone treatment (40 mg/day) resulted in a biochemical response on the 6th day and marked improvement of the biliary tree on MRCP at 1 month; however, Wirsung duct dilation persisted. (Figure 2B).

**Figure 2:**
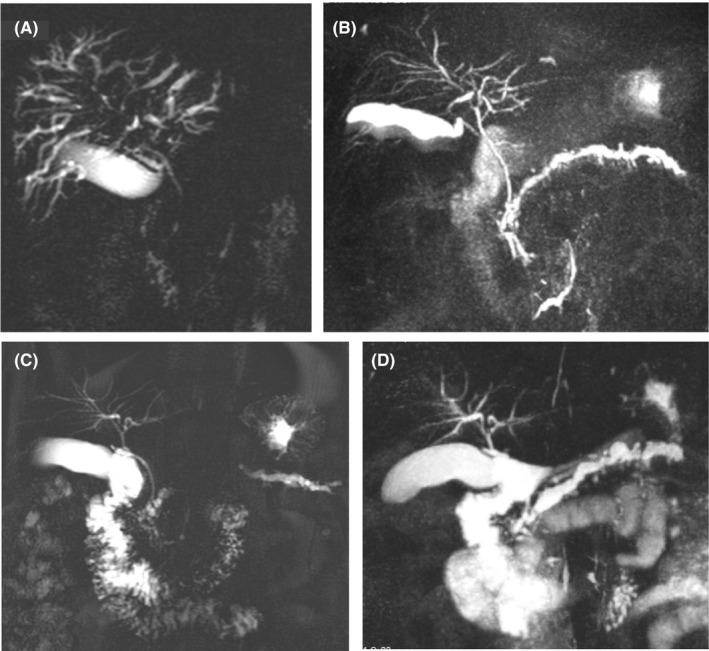
A, The MRCP image before treatment. B, The MRCP image after one month of steroid treatment. C, The MRCP image at 9 mo after the initial MRCP. D, The MRCP image at 21 mo after the initial MRCP

Liver enzyme and IgG4 levels returned to normal limits, and the steroid dose was tapered to 5 mg/day. The MRI at 9 (Figure 2C) and 21 months (Figure 2D) showed a normal biliary tree but a markedly dilated, tortuous Wirsung duct; the hypervascular mass had disappeared. The patient is receiving azathiopurine only (75 mg/day) at 41 months with normal biochemistry.

A steroid trial entails the risk of administering immunosuppression to a malignancy patient.^1^ Because our patient was not a candidate for surgery, a trial would not radically worsen his prognosis. Prevention of irreversible injury is vital.^2^ The rapid healing of the seemingly hopeless radiologic picture shows that the point of irreversibility remains to be defined. The progression of the Wirsung duct dilation is unexplained.

## CONFLICT OF INTEREST

None declared.

## AUTHOR CONTRIBUTIONS

İÖ and SK: served as the attending physicians of the patient, wrote and proof‐read the article; AP: involved in radiologic assessment, writing and proof‐reading; YS: involved in PET assessment, writing and proof‐reading.
